# ‘First, do no harm’: systematic program evaluation of an equine veterinary service-learning initiative with Indigenous communities in Canada

**DOI:** 10.1186/s12909-024-05234-3

**Published:** 2024-03-14

**Authors:** Jean-Yin Tan, Yvonne Poitras Pratt, Patricia Danyluk

**Affiliations:** University of Calgary, Calgary, Canada

**Keywords:** Program evaluation, Service-learning, Community, Veterinary, Social justice

## Abstract

**Background:**

Veterinary students have historically lacked meaningful experiential learning opportunities in equine medicine. At the same time, there are barriers to accessing veterinary care in Indigenous communities stemming from colonial injustices. In 2018–2019, a partnership was initiated where University of Calgary students began to provide equine veterinary services to Indigenous communities. As the first-documented equine veterinary service-learning initiative in Indigenous communities embedded in a veterinary curriculum, the purpose of the study is to systematically evaluate the program for its potential impact as part of a formative process for improvement.

**Methods:**

Multiple parties in the program were engaged in a convergent, parallel, mixed-methods systematic program evaluation to explore the main program outcomes: (1) equine veterinary care; (2) clinical experiential student education; (3) cultural training of veterinary professionals and students; and (4) education of community members. The hypothesis was that ethical development using the “first, do no harm principle” would lead to benefits including a healthy horse population, a technically and culturally competent veterinary community, and an educated horse clientele.

**Results:**

The program had a positive impact on accessibility to veterinary care and self-reported improvement in veterinary and cultural competency. In addition to the hypothesized program outcomes, additional program outcomes and effects were identified, including reciprocal learning and relationship building with the Indigenous community, leading to trust and equity-building. The students learned from both the in-community programming as well as the Indigenous community members they worked with.

**Conclusion:**

Program evaluation of an equine service-learning initiative in Indigenous communities reveals multiple and profound impacts including improved patient health status, wider scope of veterinary and cultural learning, strengthened relationships, and reciprocal learning with partnering Indigenous communities.

## Introduction

This study focuses on a unique equine service-learning program in southern Alberta that brings the need for hands-on equine veterinary education and the need for increased accessibility to equine veterinary care together through partnership of two First Nations communities with a faculty member in veterinary medicine. The success of the program is contingent, we argue, on understanding the need for culturally appropriate education, including social justice concepts, prior to and continuing into, program delivery.

The principle of “First, do no harm” is resident in both human and veterinary medicine, and represents a fundamental ethical tenet in the care of living beings. Reflecting a similar ethos, one of the first concerns raised by one of the co-authors, an Indigenous education scholar (YPP), was, “Are you sure you’re not doing more harm than good?” when first introduced to the concept of an equine veterinary service-learning initiative partnering with nearby Indigenous communities surrounding Calgary. Importantly, from the inception and development of this equine community practice program and clinical rotation, the program objectives have focused not only on advancing veterinary student knowledge and skills by providing education and equine veterinary care to underserved horse owners, but the program also seeks to increase professional awareness of cultural and socioeconomic contexts relative to veterinary care where the needs of Indigenous horse people are centred. This mutuality honours the core tenet of social responsibility and reflects the honouring of millennia-long Indigenous presence on these lands alongside Indigenous principles of reciprocity, relationality, and respect [1]. Notably this initiative is responsive to the University of Calgary Indigenous Strategy, the Truth and Reconciliation Commission of Canada’s Calls to Action [2], and an honouring of the United Nations Declaration on the Rights of Indigenous Peoples [3]. These local, national, and international initiatives represent a growing awareness and need for restitution stemming from colonial injustices that disadvantaged Indigenous peoples to the benefit of colonizing groups. Indigenous scholars, such as Māori educator Linda Tuhiwai Smith [4] and Mikmaw scholar Marie Battiste [5] assert this colonial history has distorted, erased, and silenced Indigenous truths allowing for what appears to be a “natural” takeover of lands and resources. The act of truth-telling is the foundation upon which future relations must be based if we are to move forward [6].

This program is the first documented equine veterinary service-learning option that takes place in Indigenous communities as part of a formal veterinary curriculum. The program has resulted in success from an institutional perspective, resulting in positive feedback and accolades from students and the veterinary community [7–9]. As the first initiative of its kind at our university, the outcomes of the program must be systematically evaluated for its potential impact on the partnering communities to achieve appropriate risk assessment [10] and to adhere to Tri-Council guidelines specific to Indigenous research. Contrary to how this risk may be thought of, we see the greatest risk residing in the possibility that students, faculty, and staff may inadvertently, or not, relay stereotypical and racist assumptions to partners without the inclusion of social justice training around the repercussions of a colonial history on Indigenous peoples that include critical self-examinations of power, privilege, and positionality by service-learners [1].

### Literature review

#### Equine veterinary curriculum

A recent survey reports high dissatisfaction among students in how well their veterinary education has prepared them for equine practice [11]. The survey showed 51.4% of 2012–16 graduating survey respondents reported they were “not at all” to “moderately satisfied” with their education [11]. The lack of meaningful experiential learning opportunities in equine medicine is largely due to the high value of equine patients alongside client attitudes towards novice animal care [11]. As a result, new graduates face significant challenges transitioning into equine practice due to lack of significant skill development and mentoring. When students have had the option of experiential learning activities, including service-learning, these have primarily focused on small animal care veterinary services [12–18]. Given these realities, new veterinary graduates face significant challenges transitioning into equine professional practice due to a lack of significant skill development and mentoring in their post-secondary training.

Equitarian work is defined as equine veterinary services or care education for the welfare of equids [19]; yet, much of the focus in this specialized area has been on animal welfare rather than veterinary student education [20]. Skills that new equine veterinary graduates are expected to perform include taking a complete medical history, developing a vaccination strategy, performing a physical examination, administering oral medication, administering intramuscular injection, explaining medical conditions to a client, maintaining medical records, managing time and resources, and formulating individual and herd wellness programs [21]. Competent entry-level veterinarians are expected to perform 51 of the 107 tasks, with a mean proficiency score of >  = 75.

At the University of Calgary Faculty of Veterinary Medicine (UCVM), an internal curriculum review and graduate exit survey from 2016 revealed that students felt equine rotations tended to be unwelcoming and hands-off [22]. Some students noted they did “not feel competent at all” in the equine field upon graduation from the UCVM veterinary program. Based on data from national veterinary organizations and concerns raised within the internal curriculum review, UCVM faculty noted a real need for high-quality hands-on equine clinical experiences in the program. The service-learning program detailed below represents an ethical and critical response to this need.

#### Critical service-learning

Service-learning, a form of experiential learning that integrates community service with academic curriculum, is a powerful teaching and learning strategy that results in improved student attitudes towards learning, civic and critical community engagement, social skills, academic performance, and self-awareness [23]. Although the practice of service-learning began as an opportunity for students to learn outside the classroom, over the years it has evolved beyond an act of volunteering to that of a critical orientation where issues of power, privilege, and positionality are considered [1, 24–27]. Critical service-learning, unlike earlier modes of service-learning, raises awareness of students around social justice concepts and historical and contemporary injustices that exist within and throughout their service-learning experience. Transformative learning [28] often results from these intense community-based experiences. In this scenario, students may encounter disorienting dilemmas that cause them to question previously held assumptions or beliefs and, in taking up tenets of critical thinking through guided reflections and inquiry, the way they think about the world is transformed.

Societal factors including poverty and geographic isolation stemming from a colonial past are some of the barriers present in accessing veterinary care in Indigenous communities, yet horses hold an important contemporary, historical, cultural, and spiritual role in Indigenous communities and cultures [19].

Partnering with Indigenous communities in a service-learning program requires special considerations, including introducing students to basic education specific to local communities, including an introduction to colonial history, the impacts of colonialism, and local protocols. Requisite time must be set aside to establish trust and build relationships with Indigenous community members prior to students spending time in community [1]. When carefully designed, community-based service-learning experiences offer students the opportunity to experience the interaction of theory and practice, cultivate relationships with community partners, and challenge hegemonic norms [29]. In examining ethical issues from a critical perspective, students further develop professional values and moral positioning which will guide their future practice [30].

This article shares findings from the first-ever equitarian project that combines student-oriented critical service-learning with the equine veterinary needs of First Nations peoples residing on reserves in Canada. While some readers may focus on the clinical service provision for clients and hands-on learning experience for students, the potential for transformative learning beyond curricular expectations driven by principles of social justice as well as learning firsthand the impacts of colonialism on Indigenous peoples living on lands now called Canada is significant.

#### Creation and development of initiative

In 2017, UCVM faculty member and primary author (JT) set out to meet the academic need for hands-on equine veterinary clinical training for students alongside a compelling need for preventative veterinary care in local Indigenous communities by initiating a partnership between UCVM and a nearby Indigenous community (Tsuut’ina First Nation). With the help of a well-respected Indigenous community member, active with horses, and serving as a community liaison, the first equine service-learning initiative was delivered in 2018 in the form of a mandatory full-day laboratory (with a choice between equine and bovine content) for all 33 second-year students within the Clinical Skills course. In the equine laboratory setting, students integrated knowledge, professional skills, and clinical skills by interacting with horse people^1^ and determining what care each equine patient needed, educating around these needs, and then providing preventive services including deworming and vaccinations.

Building on the success of this 2018 initiative, the single-day laboratory (offered to all 35 students in the second-year class) was expanded in 2019 with the addition of a two-week clinical rotation offered to four fourth-year students. Another partnership was initiated through community connections, this time with Siksika First Nations, another nearby Indigenous community. With the extended time available during the rotation and understanding the need for students to acquire cultural awareness, JT introduced cultural training with an Indigenous Elder as part of the mandatory programming for participating students.

In 2020, JT reached out to education scholars (YPP and PD), who have conducted research around critical service-learning in educational settings involving Indigenous communities. Working together, we expanded the cultural training in the rotation to include mandatory readings and expanded mandatory educational seminars for both students and faculty/staff involved in the rotation. After additional consideration of the risks and benefits of bringing large numbers of students not trained in cultural competence (due to time limitations) and not necessarily interested in veterinary service-learning opportunities with First Nations partners, the one-day clinical skills laboratory was discontinued in 2020.

The public health restrictions associated with the COVID-19 pandemic also significantly impacted our plans and programming was canceled with short notice in 2020 and 2021. Essential services, however, continued to be provided by JT for continuity of care and to honour the commitment made to Indigenous communities. As a result, program evaluation for this study focused on the clinical rotation offered in 2022, which involved four students, three faculty members, three technicians, and one technician assistant. The learning outcomes of the rotation included: providing equine veterinary care to underserved horse populations in Indigenous communities; integrating knowledge, clinical skills, professional skills, and clinical reasoning in a clinical environment led directly by faculty members; improving awareness of cultural and socioeconomic contexts, including colonial impacts, as relevant to provision of veterinary care; and, educating Indigenous community members on equine health care.

#### Program evaluation in complex settings

Program evaluation is the “systematic assessment of programs designed to improve social conditions and our individual and collective well-being” [31] and is commonly utilized to determine the impact and quality of initiatives impacting health outcomes for individuals and communities [32]. Well-intentioned social programs do not necessarily lead to better outcomes and sometimes even lead to unanticipated negative repercussions, since problems that social programs aim to solve tend to be complex ignoring wider systemic issues such as colonialism [31]. An effective implementation strategy is essential to meet the social justice intention of the program.

The purpose of program evaluation is to determine the overall worth of an education program and to plan program improvement [33]. A formative program evaluation is created with the goals of timely and useful findings that can be immediately used to inform the refinement of the program [34]. Impact evaluation asks the question of whether desired outcomes are achieved and whether unintended negative side effects have occurred in the process and is suitable for any program intended to bring about change [31]. This type of evaluation estimates the net effects of a program on multiple parties and in the process, measures if the program is well implemented in achieving its stated goals. Multiple dimensions of outcomes and multiple measures of outcomes are used to ensure a comprehensive approach that does not leave out relevant outcome dimensions [31]. Data for program outcomes include observations, records, responses to surveys and interviews, and physical measurement. Program impact refers to the difference between outcomes with project implementation and what would have occurred otherwise, therefore dealing with cause-and-effect relationships, and aims to determine if desired effects were achieved and what the magnitude of the change was [31]. Program impact theory is often depicted via causal diagrams that visually show the cause-and-effect linkages between program activities and expected outcomes, ie. following a chain of events from program action to proximal (direct and immediate), intermediate, and distal (ultimate) outcomes.

## Methods

Prior to commencing the study, the researchers gained ethics approval from the institution’s Conjoint Health Research Ethics Board (Protocol No. REB20-1819), aligning with institutional requirements. The objective of this study was to systematically evaluate an equine veterinary service-learning initiative taking place in two Indigenous communities from both an Indigenous and institutional lens. A formative impact evaluation on this new initiative was conducted to examine the experiences of faculty and staff, students, horse people, and patients in the program and to ensure the continuous improvement of the program.

**Fig. 1:**
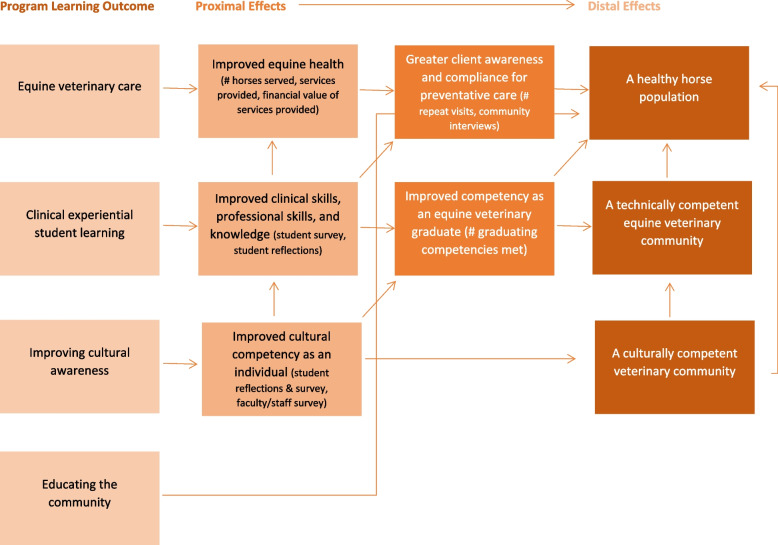
Program impact theory showing expected program effects on proximal and distal Outcomes

Systematic formative program evaluation took place during the clinical rotation in August 2022 to assess the impact of the program to-date. The two-week clinical rotation consisted of eight days in Indigenous reserves providing clinical services and two days at the university in training and discussion. The initiative was led by one faculty member and two technicians, with the assistance of two other faculty members, one other technician, and one technician assistant as needed. Four veterinary students were enrolled in the rotation. Multiple partners and parties involved in the program (faculty members, technical staff, students, and horse people) were engaged in a convergent, parallel, mixed-methods evaluation to explore the four main hypothesized outcomes of the program (Fig. 1): equine veterinary care, clinical experiential student learning, improving cultural awareness, and educating the community.

The first hypothesized program learning outcome of equine veterinary care was evaluated by quantifying the horses served, the services provided, and the financial value of veterinary services. The convergent parallel mixed-methods approach lent itself to the temporal nature of the work where data was simultaneously collected and equally prioritized within a two-week clinical rotation, then analyzed separately after the rotation and results merged and integrated to provide comprehensive results that thoroughly explored the impact of the program [35]. The purpose of the convergent design was to “obtain different but complementary data on the same topic” to triangulate the information [36].

The second hypothesized program learning outcome of clinical experiential student learning was evaluated through a student pre-rotation and post-rotation survey. Veterinary students taking the clinical rotation were invited to participate in the study by a research assistant who was not involved in the rotation or in their grading. Students who participated in the research provided written consent and were asked to complete a questionnaire at the beginning and then at the end of the clinical rotation and invited to complete multiple reflections throughout the rotation. The students were assured that their decision to participate in the study and their performance on the reflections would not affect their grade for the clinical rotation. One section of the questionnaire focused on evaluating cultural learning while the second section evaluated the learning outcomes from the rotation (Table 1). The self-evaluation questions on general knowledge and skills and perception towards social responsibility and engagement were adapted from Van Patten’s research [18]. The instructions for the student reflections were to write a reflection a minimum of four times throughout the rotation and whenever they felt significant learning had taken place. Optional guiding questions included: What was your most significant veterinary learning today? What was your most significant cultural learning today? What connections have you made between your learning and the cultural training sessions? How do you see yourself implicated in the Truth and Reconciliation Commission calls to action, relative to the veterinary medical practitioner role you are preparing yourself for? What do you still need to learn more about? Student learning and experience was assessed by comparing the number of competencies achieved relative to the 107 equine-related competencies expected of a new graduate [37].

**Table 1:** Pre-rotation questionnaire completed by students

Cultural Perspectives*	Social Responsibility**	Additional Comments or Thoughts
1.I think my beliefs and attitudes are influenced by my culture 2.Veterinary professionals’ own cultural beliefs influence veterinary care decisions 3.Time in the veterinary curriculum devoted to the promotion of student self-awareness and well-being is time well spent 4.A veterinary professional’s ability to communicate with clients is as important as his/her ability to solve clinical problems 5.The presence of more than two family members or agents for the horse owner is disruptive to veterinary care and should be prohibited 6.The quality of patient care could possibly be compromised if a veterinary professional is oblivious to the family’s cultural attributes and values 7.As a veterinary professional if I needed more information about a person’s culture to provide a service, I would feel comfortable asking the person or one of their family members 8.Indigenous peoples, due to their own cultural beliefs and values, have the poorest animal health status in Canada 9.Indigenous peoples should take more individual responsibility for improving their own animals’ health 10.The Western medical model is sufficient in meeting the health needs of all animals including those owned by Indigenous peoples 11.All Canadians need to understand Indigenous history and culture 12.Indigenous peoples should not have to change their culture just to fit in 13.We practice equity in the provision of veterinary care by treating Aboriginal people the same as all other clients 14.I need to think beyond the individual when considering Indigenous veterinary care 15.I have a social responsibility to work for changes in Indigenous veterinary care	1.I am motivated to partake in services intended to help people that are disadvantaged 2.I am motivated to volunteer within the animal health community 3.I feel a strong sense of social responsibility 4.I am comfortable working with marginalized populations 5.Vets should provide free services for Indigenous populations 6.People who cannot afford veterinary care for their pets should not own them 7.I believe vets can impact human health 8.It frustrates me when people cannot afford to pay for their pet’s health care 9.I plan on being involved with community outreach after graduation	

^*^Adapted from Ryder et al. (2017)** Adapted from Van Patten et al. (2021)

Improving cultural awareness of veterinary professionals was the third hypothesized program learning outcome. This was evaluated using the aforementioned pre-rotation and post-rotation survey (Table 1), a portion of which was adapted from a validated questionnaire used in a similar study to measure attitude change in health professionals in Australia after completing an Aboriginal health and cultural safety training programme [38]. This was used to assess cultural learning by students, faculty members, and staff. Individuals participating in these programs completed the questionnaire on Day One prior to any cultural training seminars or service-learning experiences and completed the same questionnaire on the final day at the completion of the clinical rotation. Faculty and staff participants were recruited by the research assistant, who obtained written consent for their participation in the research by filling out a similar pre- and post-rotation questionnaire and attending two cultural awareness workshops—each led by Indigenous and allied professionals—at the beginning of the rotation. The faculty member who led the rotation and authored this study was excluded from participation and from the data set. Some faculty and staff participants had participated in the first rotation three years ago, yet cultural training at that time was less rigorous and not mandatory. Student reflections were also used to assess changes in cultural competency.

The hypothesized fourth program learning outcome of educating the community was evaluated using community interviews. During the clinical rotation, all Indigenous horse persons were approached to gauge their interest in study involvement by an independent research assistant. A small financial incentive was offered for their participation and this data helped evaluate the initiative and develop future directions for the initiative from a community perspective. The research assistant, who had previous research experience with Indigenous communities, conducted all semi-structured interviews, which included the following questions:

•*What kinds of things do you want students to know before they come into your community to work with the horses? How do you think they should learn about this?*
*•Overall, tell me how happy or unhappy you have been with the services you received from the vet students.*
*•Did your horses see a vet every year before this started? How have things changed for you and your horses since the services started?*
*•What was the best thing about having the vet students provide service to your community?*
*•What would you like to see changed if this service was offered again in the future?*
*•Would you like to see the vet services expand? What would be the ideal case?*
*•If there were vet services regularly available for small animals, horses, or cows in Calgary would you use it? Which one(s) would you use? What if there was a fee associated with it? Would transportation be an issue?*
*•Are there any final thoughts or questions you’d like to ask me before we finish?*

The interviews were recorded and transcribed, and identification of individual community members was disclosed only with permission or kept anonymous according to their wishes.

The quantitative thread of the convergent parallel mixed-methods evaluation consisted of quantification of services provided (financial and numerical) and statistical analysis of survey data. Despite the small number of participants, analysis of the pre- and post-program questionnaires completed by students, faculty members, and staff, was performed by evaluating reliability (Cronbach’s alpha) and comparing pre- and post-rotation results for significant differences using paired samples t-testing and a *p*-value of 0.05 and *p*-value of 0.10. Descriptive statistics including mean difference, standard deviation, standard error of means, and confidence intervals, were also included. The quantitative analysis provides a measure of reliability but not generalizability.

The qualitative thread of the mixed-methods evaluation consisted of student reflections and transcribed community interviews, which were qualitatively analyzed using a five-step method of thematic analysis [39]. Three researchers independently familiarized themselves with the data, generated initial codes, searched for themes, then met to review and name themes, and agreed on findings through consensus prior to producing the report. Researchers used the philosophical phenomenological method described by Giorgi [40]. In this approach, the essence of what community members shared was left intact and held as truth and, given that we were working with an Indigenous population, this approach was deemed culturally appropriate.

Finally, program evaluation findings were compared to the causal diagram created using program impact theory, to assess if outcomes lead to anticipated societal benefits and if there are any unintended negative consequences resulting from the initiatives. Using program impact theory, it was hypothesized that when ethically developed using the “first, do no harm principle,” the existing equine veterinary service-learning initiative produces a multitude of direct benefits that ultimately lead to a healthy horse population and a more technically and culturally competent veterinary community. The hypothesized cause-and-effect linkages from the course learning outcomes are depicted in a causal diagram (Fig. 1).

Findings are expected to contribute to the ongoing improvement of the program and to help inform the ethical, and socially responsible development of similar programs to address the equine veterinary educational gap in other North American veterinary schools.

## Results

### Equine veterinary care

At the time of program evaluation, the described initiative had been running from 2018 to 2022 (2018 as a single-day laboratory, 2019 as a laboratory and 2-week rotation, 2020–2021 rotations were canceled yet essential services provided, and the rotation resumed in 2022). Cumulatively, the program served 50 horse people and 212 horses, providing approximately $136,000 [CAD] worth of veterinary services. The 2022 rotation alone provided veterinary services to 71 horses. This includes 31 horses used in a community after-school riding program that runs 5 days per week at Tsuut’ina Nation over the past 17 years [41].

Veterinary services have focused on preventive care, including annual physical examinations, deworming, vaccinations, and advanced routine services such as lameness, dentistry, anesthesia, and castration. Many rich clinical learning experiences have occurred during the rotation that extend beyond routine services, including respiratory diagnostic testing (eg. bronchoalveolar lavage), laceration repair, skull fracture management, and wound revision surgery. Altogether, over 1100 vaccines and over 250 doses of dewormer were administered. During the clinical rotations offered in 2019 and 2022, 8 students performed over 100 dental flotations and approximately 40 full lameness examinations, including diagnostics and therapeutics.

### Clinical experiential student learning

At UCVM, students receive 49 weeks of practical hands-on clinical rotations during their final year in the program. This two-week rotation is offered as an elective within the overall program span of 49 weeks for any veterinary student regardless of area of emphasis. There are no prerequisites for enrolment and 4 students elected to take the rotation in 2022.

Students gained experience with basic procedures [21] and more advanced skills: estimating weight, interpreting complete blood counts, serum biochemical profiles, and endocrinology testing, managing common ailments (corneal ulcers, wounds), performing venipuncture and placing intravenous catheters, flushing nasolacrimal ducts, performing fecal flotations, performing anesthesia, taking radiographs, explaining parasite management, medical conditions, and sharing discharge instructions with clients, working with a veterinary team, and performing dentistry, lameness examinations, and castrations. In the 2-week rotation, students were able to practice 65 of the total 107 competencies listed as requirements [21, 37]. This number represents 61% of the competencies expected of a graduating veterinarian (Table 2). Students were evaluated by supervising veterinarians using in-training evaluation reports to rate their skills using Likert scales for competency. Competent entry-level veterinarians are expected to perform 51 of the tasks, with a mean proficiency score of >  = 75 [21]; with 67 skills achieved, our students exceeded this professional expectation.

**Table 2:** Competencies covered by the rotation

Medical knowledge (10 of 14 skills satisfied) *Take a complete medical history *Estimate weight *Interpret a hemogram (CBC) *Interpret a serum biochemical profile *Identify the risk factors associated with metabolic syndrome and be able to discuss a complete management plan *Develop a vaccination strategy for different ages, locations and exposure risks *Recognize and treat systemic and localized pain Estimate degree of dehydration and develop an appropriate treatment plan *Analyze the basic components of a ration and make general recommendations based on the patient’s caloric needs *Understand/be able to find information on drug withdrawal periods for performance horses and have knowledge of their implications on treatment options *Develop a plan to medically manage a superficial or deep corneal ulcer, stromal abscess, or foreign body Recognize when surgical intervention is necessary for a corneal infection or injury Recognize when surgical intervention is necessary for a corneal infection or injury Recognize and develop a treatment plan to manage Equine Recurrent Uveitis Discuss testing for genetic diseases and coat colors Examination (2 of 6 skills satisfied) Perform a physical examination on a foal and an adult horse Auscult and interpret respiratory sounds of foals and adults *Auscult and interpret heart sounds *Auscult and interpret gastrointestinal sounds Perform a neonatal examination Perform a post-foaling examination Techniques (13 of 23 skills satisfied) *Administer medication PO (paste) *Administer an IM injection *Perform jugular venipuncture *Administer an IV injection *Administer a SC injection (medication or fluids) Use an HCT tube and tabletop centrifuge to determine PCV concentration Use a refractometer to determine total plasma protein concentration Use a portable machine to determine glucose and lactate concentrations in fresh blood Recognize overt lipids in a venous blood sample *Place and secure a catheter in the jugular vein *Pass a nasogastric tube *Administer liquid medication with a nasogastric tube *Perform fluorescein staining of the cornea Perform and interpret a test for measuring IgG concentration in foals Perform an enema Perform a skin scraping Aspirate a mass *Flush a nasolacrimal duct *Perform a fecal flotation Prepare and read a Gram stained slide *Perform an auriculopalpebral nerve block *Perform a complete ophthalmic examination with direct or indirect ophthalmology	Obtain measurements of IOP Reproduction (0 of 8 skills satisfied) Place a tail wrap on a mare Perform rectal palpation for a mid- and late-stage Pregnancy Examine the cervix with a vaginal speculum Obtain a uterine sample for culture Know the basic indications and technique for performing a Caslick’s procedure Discuss the basic techniques of breeding a mare with CSS, and frozen semen Discuss the process of collection of semen for the purpose of developing a plan to breed a client’s mare Discuss the aspects of an unsuccessful breeding cycle Anesthesia (5 of 6 skills satisfied) *Sedate an adult horse *Perform a line block for local anesthesia Sedate a foal *Monitor the depth of anesthesia *Develop an anesthesia plan for both long and short procedures requiring general anesthesia *Develop a sedation plan for long-term standing procedures Husbandry techniques (6 of 8 skills satisfied) *Apply a lip twitch *Approach, capture, and attach a halter and lead *Elevate and examine a hindlimb *Elevate and examine a forelimb *Perform proper animal restraint *Tie a quick-release knot Restrain a foal Apply a tail tie Surgery (6 of 14 skills satisfied) *Apply a wound dressing *Apply a bandage to a limb *Apply a bandage to a foot Remove sutures and staples from a healed surgical wound *Clean and debride a wound Suture proficiently *Perform surgery: properly gown and glove and aseptically prepare a patient for surgery Perform hemostasis; isolate, clamp, and use hand and instrument ties to ligate a bleeding vessel *Demonstrate proper postoperative patient care including wound evaluation Properly prepare and sterilize a surgical pack Know the basic principles of wound management and appropriate use of drains for a soft tissue lesion Identify, diagnose with radiographs, and create an appropriate treatment plan for a sinus infection Determine the need for and interpret radiographs of the neck Discuss and understand the basic technique for performing and interpreting a myelogram Client education and regulatory (5 of 6 skills satisfied) Complete a health certificate *Explain gastrointestinal parasite identification, treatment, and prevention *Write clear discharge instructions in lay terminology *Explain medical conditions to a client clearly and	concisely in an understandable manner *Explain proper healthy animal nutrition to client *Recognize the risk factors of age-related issues in older horses and discuss preventative geriatric monitoring Business of veterinary medicine (4 of 6 skills satisfied) *Maintain complete and concise medical records Effectively communicate over the telephone *Recognize major breeds, colors and performance disciplines by appearance *Manage time and resources appropriately to meet the needs of a busy schedule while covering unanticipated aspects of the scheduling *Demonstrate a willingness to work cooperatively as a member of a mixed team of professionals and lay coworkers Communicate effectively (oral and written) with referral veterinarians and referring veterinarians Dentistry (5 of 7 skills satisfied) * Know the dental formula and eruption schedule and how to determine approximate age from the dentition *Understand the anatomy and physiology of the teeth and oral cavity and associated structures *Perform and document a complete oral and dental examination *Recognize common dental and oral pathology *Perform dentistry procedures to manage sharp enamel points and occlusal abnormalities Understand the management of persistent or mal- erupted deciduous teeth Discuss the indications and locations of local and regional nerve blocks associated with dentistry Lameness (7 of 9 skills satisfied) *Obtain a history for a horse presented for a lameness/performance issue *Demonstrate proficient use of hoof testers Locate and treat a subsolar abscess, with guidance if needed *Perform a musculoskeletal exam of a horse presented for lameness issues *Perform a lameness examination and determine degree of lameness and be able to identify the affected limb *Discuss a diagnostic plan for a horse presented for lameness *Perform basic lower limb diagnostic nerve blocks *Discuss the indications and locations of advanced diagnostic nerve blocks Identify angular limb/flexural deformities in foals and discuss options for correction Total: 65/104 competencies satisfied by the rotation

### Improving cultural awareness

#### Student questionnaires

There was a 100% response rate (n = 4) to the student questionnaires, both pre- and post-rotation. Due to the small sample size, reliability was low (Cronbach’s alpha = 0.38 and 0.54 pre- and post-rotation, respectively) with calculated power of 0.11. Post hoc statistical power analysis demonstrates that a total sample size of 34 participants is required to achieve the ideal minimum power of 0.80.

Paired samples t-testing on student surveys filled out before and after the rotation revealed no significant differences using a cut-off of *p*-value < 0.05 (Table 3). Using a cut-off value of <  = 0.10, there was a significant difference between pre-training scores (M = 3.00, SD = 0.82) and post-training scores (M = 2.00, SD = 0.82;[(t(3) = 2.45, *p* = 0.09]) with a large effect size (Hedge's g = 1.06) for the question “people who cannot afford veterinary care for their animals should not own them” (*p* = 0.09, mean difference = 1.00). This indicates that after the rotation, students tended to change their mind in favour of all those owning animals being able to access veterinary care, regardless of financial status. One comment on the post-rotation survey noted that veterinarians should not carry the burden of providing no/low-cost veterinary care alone and they should work together with the communities, the government, and non-Indigenous Canadians to support essential needs for veterinary care.

**Table 3:** Paired-samples t-test, pre- and post- rotation question results, from student survey

Question	Mean Difference	SD	SEM	95% CI: lower	95% CI: upper	t	df	*p* (2-tailed test)
1	.25	.96	.48	-1.27	1.77	.52	3	.64
2	-.50	1.91	.96	-3.55	2.55	-.52	3	.64
3	-.25	.50	.25	-1.05	.55	-1.00	3	.39
4^a^	–	–	–	–	–	–	-	–
5	.25	.50	.25	-.55	1.05	1.00	3	.39
6	-.50	.58	.29	-1.42	.42	-1.73	3	.18
7	.20	1.71	.85	-2.47	2.97	.29	3	.79
8	.50	1.00	.50	-1.09	2.09	1.00	3	.39
9	-.50	1.00	.50	-2.09	1.09	-1.00	3	.39
10	.25	.96	.48	-1.27	1.77	.52	3	.64
11^a^	–	–	–	–	–	–	-	–
12^a^	–	–	–	–	–	–	-	–
13	-.25	.50	.25	-1.05	.55	-1.00	3	.39
14	.25	1.26	.63	-1.75	2.25	.40	3	.72
15^a^	–	–	–	–	–	–	-	–
16^a^	–	–	–	–	–	–	-	–
17	-.50	.58	.29	-1.42	.42	-1.73	3	.18
18	-.50	.58	.29	-1.42	.42	-1.73	3	.18
19	.00	.82	.41	-1.30	1.30	.00	3	1.00
20	.00	.82	.41	-1.30	1.30	00	3	1.00
21	-.50	1.29	.65	-2.55	1.55	-.78	3	.50
22	.00	.82	.41	-1.30	1.30	.00	3	1.00
23	.25	.50	.25	-.55	1.05	1.00	3	.39
24	.25	.50	.25	-.55	1.05	1.00	3	.39
25	-.25	.50	.25	-1.05	.55	-1.00	3	.39
26	-.50	1.00	.50	-2.09	1.09	-1.00	3	.39
27	-.25	1.50	.75	-2.64	2.14	-.33	3	.76
28	1.00	.82	.41	-.30	.30	2.45	3	.09
29^a^	–	–	–	–	–	–	-	–
30	.25	.50	.25	-.55	1.05	1.00	3	.39
31	.25	.50	.25	-.55	1.05	1.00	3	.39

*SEM* standard error mean

^a^Unable to calculate paired-samples t-test because the standard error of the difference is 0

Descriptive statistics (Table 4) revealed high student satisfaction with the rotation, with mean scores of 5.0/5.0 on the items “the initiative is a worthy use of my time,” and “this initiative is an important part of the curriculum,” and “if available, I would participate in this type of initiative again.” Lower ratings were associated with the statement that the initiative should be mandatory for all students.

**Table 4:** Paired-samples t-test, pre- and post-rotation question results from faculty and staff survey

Question	Mean Difference	SD	SEM	95% CI: lower	95% CI: upper	t	df	p (2-tailed test)
1	.80	.84	.37	-.24	1.84	2.14	4	.10
2	.20	.84	.37	-.84	1.24	.54	4	.62
3	-.40	.55	.24	-1.08	.28	-1.63	4	.18
4	.60	1.52	.68	-1.28	2.48	.89	4	.43
5	.00	.71	.32	-.88	.88	.00	4	1.00
6	.40	.55	.24	-.28	1.08	1.63	4	.18
7	-.20	1.30	.58	-1.82	1.42	-.34	4	.75
8	-.20	1.64	.73	-2.24	1.84	-.27	4	.80
9	.20	1.10	.49	-1.16	1.56	.41	4	.70
10	.40	.89	.40	-.71	1.51	1.00	4	.37
11^a^	–	–	–	–	–	–	–	–
12	-.40	1.14	.51	-1.82	1.02	-.78	4	.48
13	.80	.84	.37	-.34	1.84	2.14	4	.10
14	.00	1.00	.45	-1.24	1.24	.00	4	1.00
15	1.00	1.41	.63	-.76	2.76	1.58	4	.19
16	.60	.89	.40	-.51	1.71	1.50	4	.21
17	.40	1.14	.51	-1.02	1.82	.78	4	.48
18	.00	.71	.32	-.88	.88	.00	4	1.00
19	.60	1.14	.51	-.82	2.02	1.18	4	.31
20	.40	.55	.24	-.28	1.08	1.63	4	.18
21	.60	1.95	.87	-1.82	3.02	.67	4	.53
22	.00	.71	.32	-.88	.88	.00	4	1.00
23	-.20	1.64	.73	-2.24	1.84	-.27	4	.80
24	-1.40	3.36	1.50	-5.57	2.77	-.93	4	.40

*SEM* standard error mean

^a^Unable to calculate paired-samples t-test because the standard error of the difference is 0

#### University faculty and staff questionnaires

In addition to evaluating the students’ perspective on their cultural competency before and after the rotation, questionnaires were also distributed to faculty and staff involved in the clinical rotation. The response rate from university faculty and staff was 71% (*n* = 5). Reliability was moderate (Cronbach’s alpha 0.71 pre-rotation and 0.67 post-rotation), likely due to small sample size with calculated power of 0.14. Total sample size required to achieve 0.80 power is 34 participants.

Paired-samples t-test (Table 5) revealed no significant differences in pre- and post-rotation survey responses using a cut-off *p*-value of 0.05. However, 2 questions yielded *p* <  = 0.10: Question 1 “I think my beliefs and attitudes are influenced by my culture” (t(4) = 2.14, *p* < 0.10) and Question 13 “we practice equity in the provision of veterinary care by treating Aboriginal people the same as all other clients” (t(4) = 2.14, *p* < 0.10). There was a shift in the post-rotation survey towards disagreement with the statements that beliefs and attitudes are influenced by culture and that we practice equity in treating Aboriginal people the same as other clients. No comments were provided by faculty/staff to further elucidate these beliefs.

**Table 5:** Descriptive statistics, evaluation of the initiative, student survey

Question	N	Min	Max	Mean	SD
The initiative is a worthy use of my time	4	5.00	5.00	5.00	.00
This initiative is an important part of the curriculum	4	5.00	5.00	5.00	.00
If available, I would participate in this type of initiative again	4	5.00	5.00	5.00	.00
The length and depth of the initiative was sufficient for my learning	4	4.00	5.00	4.25	.50
This initiative should be mandatory for all UCVM veterinary students	4	3.00	5.00	3.75	.96

Descriptive statistics showed high ratings relative to the rotation (Table 5), but lower agreement with the statements that the length and depth of the initiative was sufficient. There was little support for the idea that the initiative should be mandatory for all students.

When instructor data was grouped with students, there was a significant change in responses to questions of perception towards social responsibility and engagement when using a cutoff of *p*-value 0.10 (Table 6; *p* = 0.09). However, there was no difference in survey results for faculty, staff, and students as a whole (Table 7).

**Table 6:** Paired-samples t-test, pre- and post-rotation question category results

Category	Mean Difference	SD	SEM	95% CI: lower	95% CI: upper	t	df	*p* (2-tailed test)
Faculty and Staff								
Cultural perspectives	3.20	5.36	2.40	-3.45	9.85	1.34	4	.25
Perception towards social responsibility and engagement	1.00	7.31	3.27	-8.08	10.08	.31	4	.78
Students								
Cultural perspectives	-.25	4.99	2.50	-8.19	7.69	-.10	3	.93
General knowledge and skills	-1.50	2.89	1.44	-6.09	3.09	-1.04	3	.34
Perception towards social responsibility and engagement	1.00	.82	.41	-.30	2.30	2.45	3	.09

*SEM* standard error mean

**Table 7:** Paired-samples t-test, pre- and post-rotation overall survey results

Survey	Mean Difference	SD		SEM	95% CI: lower	95% CI: upper	t	df	*p* (2-tailed test)
Faculty and Staff	4.20	11.50		5.14	-10.08	18.48	.82	4	.46
Student	-.75	6.60		3.30	-11.25	9.75	-.23	2	.84

*SEM* standard error mean

#### Student reflections

From a qualitative perspective, the student reflections revealed four themes: valuing of Indigenous worldviews; gaining a deeper understanding of the impacts of colonialism and systemic racism; gaining increased awareness of the relationship between systemic racism and veterinary medicine; and, finally, a recognition of the importance of taking personal responsibility for building authentic relationships with Indigenous community members.

### Valuing indigenous worldviews

Veterinary students gained insights into Indigenous worldviews and beliefs in the community rotations, including community beliefs surrounding the origins of horses and their significance within the specific community and culture. One student shared that Indigenous knowledge challenged “the idea that the horse was absent in North America before colonizers arrived, and that they lived in what is now called North America much before colonization. Their histories speak of how horses lived in close harmony with Native peoples.” Students developed an understanding of the spiritual relationship between people (two-legged) and horses (four-legged) as one of equal standing where the horse is considered on par to humans, as a relative. Students described Indigenous community members as “some of the most intuitive and natural horsemen I have ever met.” Several students referred to the ways in which relay races such as those at the Calgary Stampede celebrate the significance of horses to Indigenous peoples. One student noted, “We were fortunate to watch a video of the Siksika Indian relay races, which was mind-boggling. Truthfully, I hadn’t realized what was involved in this sport while working on the Thoroughbred horses, and watching these videos made me appreciate what incredible athletes these horses are.” Here, cultural connections and appreciation of Indigenous ways are being relayed by the students.

While not part of the intended learning outcomes, students noted how much they were learning from Indigenous peoples with horses while they were providing veterinary services. This reciprocal learning was evident in one student comment, “On several occasions, they (Indigenous people) stopped to show me the horse handling they use when dealing with a nervous horse to create a less stressful environment.”

### Impacts of colonialism and racism

Students learned that the federal government did not honour the original terms of Treaty 7 signed in 1877 by re-designating almost half of the lands promised to the Siksika Nation to incoming settlers. These lands are some of the most productive and mineral-rich lands in Treaty 7 and represent a great loss to Indigenous communities [41]. Students were shocked by this reality noting, “Recently, the federal government provided a settlement to the Siksika Nation in the form of money in repayment of stolen land. Until this rotation, I was completely unaware,” and “it astounds me that one of the largest land settlements happened within minutes of Calgary and I had no idea.”

Another student reflected on how a colonial society and education system either taught them little about Indigenous knowledges or devalued Indigenous ways. The importance of deep and active listening in unlearning colonial and hegemonic ideas was obvious in their response.

These two weeks were nothing short of subtle moments, shared connection, and quiet education that in direct and indirect ways challenged the colonial ideas that

are still lingering in how I see the world.

Every day on reservation [sic] I had the chance to meet with community members.

and listen. And honestly, I think that is a huge part of decolonization, truth,

and reconciliation. Listening. Indigenous stories, voices, and languages.

have been silenced for generations and it’s time they are given the mike [sic]. It is hard to hear these stories and not want to do everything you can to fix it. It is hard

to hear these stories and not get somewhat defensive. It is hard to hear these stories and listen to what is said. We all can hear, but it is another thing to listen.

These reflections demonstrate students are starting to acknowledge the insidious nature of colonialism and systemic racism that is hegemonically positioned as the norm in our contemporary lived experiences.

### Systemic racism in and beyond the practice of veterinary medicine

Student experiences during rotations led to examinations of personal complicity. One student commented, “I wish that my own beliefs about Indigenous communities and individuals did not have a history of colonial, racist, and white saviour undertones. But they did.” This type of growing awareness was also evident in another student comment on how systemic racism continues to benefit non-Indigenous peoples, “I am here now, on stolen land, speaking a language that is not native to this place, and benefiting from the colonial actions of Canada’s past.” Another student shared, “I was humbled by my ignorance, angered at the injustice, and saddened that so little has been done to make things right.” Another student described their learning as an “uncomfortable education…it has been and will continue to be a fruitful and uncomfortable journey of decolonizing my worldview.” Each student recognized the ways in which systemic racism continues to benefit the colonizers and disadvantage Indigenous peoples.

During the pandemic, requisite health restrictions exacerbated the ever-present systemic racism surrounding on-reserve care of horses in Indigenous communities as services became even more limited [9, 42, 43]. One student noted a general reluctance by veterinary service providers to provide services to Indigenous nations, “Many local Indigenous communities were denied veterinary care during the height of the pandemic, due to misconceptions and stereotypes. Even today, many of the Indigenous communities do not have access to general veterinary care for their horses and are only seen by equine veterinarians in emergencies.” Another student verbalized more truths, “Numerous times throughout the rotation we discussed how many Indigenous people are underserved by the veterinary community and in some cases even denied veterinary care.” One student noted the importance of preventative care in their role as future veterinarians, especially in light of underserved communities.

This rotation has also taught me how valuable veterinary medicine is for the horses of these Indigenous communities. Although many of these horses are regularly evaluated by veterinarians to travel across the US border for events, many have not received preventative general care such as vaccines, deworming or dental examinations in years, if ever. As we have learned throughout our education, preventative medicine is an important aspect to equine health, and is something we must advocate for as veterinary professionals.

Another student reflected on the ways in which Indigenous knowledges are ignored or devalued by professions, including veterinary medicine, stating,

There are many aspects of our society that carry inherent colonial and racist bias, science being one of them. In many scientific fields, traditional oral stories and accounts of Indigenous peoples are not recognized. The belief around the origin of horses in the Americas may seem trivial. But it is a representation of the blatant disregard for the Indigenous perspective [sic] in so much of our worldview.

This type of uncomfortable learning prompted students to commit to recognizing and confronting systemic racism, and to understand their own role in dismantling societal injustices.

I was reminded today that to avoid the conversations is in a way complicitly allowing for the perpetuation of colonial and racist perspectives. I was reminded today that these conversations can happen with grace, kindness, and compassion. I was reminded that it is my responsibility to challenge racism, stereotypes, and falsities whenever I get the chance. I was reminded that if I want to be a part of truth and reconciliation (then) I must speak up and out and not stay silent.

### Building relationships with and learning from Indigenous community members.

Students made important connections between the valuing of Indigenous knowledges and the provision of veterinary services. One student asserted, “I think that as a veterinary professional, the cultural beliefs of our clients are very important in the decision-making process.” Students expressed their wish to continue to learn from Indigenous horse people on topics including “traditional Indigenous medicine, horsemanship, and culture.” This enhanced appreciation for Indigenous worldviews led students to reflect on how colonialism and racism continues to impact the lives of Indigenous peoples, “I think it is very important to expose myself to more Indigenous cultures and perspectives, as it is not something that I, a non-Indigenous person, was exposed to growing up. I recognize this flaw in my education and would love to learn more.” This commitment to increased learning is ultimately reliant on Indigenous peoples’ willingness to share.

### Veterinary learning

In addition to cultural insights, student reflections discussed appreciation for the importance of adaptability when applying clinical skills learned in earlier years to patients during clinical rotations (“equine castrations in dorsal recumbency…isn’t [sic] always possible in a field situation”) and the importance of being thorough and not jumping to conclusions (e.g. lameness attributed to a different source than the historical diagnosis). Students also discussed their newfound confidence and comfort level in completing a patient evaluation and treatment from beginning to end, due to the repetitive nature of practicing numerous dental procedures under the guidance of supervising veterinarians.

### Educating the community

A total of 22 Indigenous horse people chose to participate in the semi-structured community interviews. An anticipated program learning outcome was to educate the community to improve client awareness and compliance surrounding preventative veterinary care. This one-dimensional outcome that centers Eurocentric ideology was challenged and enriched by engaging in discourse with Indigenous community members and further elaborating on findings from student reflections. A total of five themes were identified in the qualitative analysis of Indigenous community members’ interviews. These themes include: i) appreciation for the community-based veterinary services; ii) acknowledgement of the researcher’s commitment and relational ways; iii) reciprocal learning; iv) transportation as an issue; and v) plans for the future.

### Appreciation for community-based veterinary services

The participating Indigenous community members in this study expressed much appreciation for the program, with almost all the interviewees expressing gratitude for the provision of veterinary services. “Oh, this is a great thing for horses around here …for a vet to come out here to [care for] our horses.” Several expressed feeling a sense of relief in knowing their horses were receiving quality care and being seen on a regular basis. One said, “I think everybody is very appreciative and very happy to get the stuff done…like we see, we look out, [but] you don't [always] notice that some of the lameness or some of these things are causing a long-term injury for their horses or anything like that. And so…it's really beneficial that they do come up and do this [service].” Another horse person commented on the way in which the care lengthened the lives of the horses, “you could tell that it added years to their lives. Yeah, it's made [the horses] really come around, made them better horses.”

Several horse people commented on the respectful ways of the researcher/clinician and the students. Specifically, they noted university personnel took the time to explain what they were doing and actively listened to the horse owners' concerns. This extra time spent explaining the process to members meant “it was a natural trust after the first time they told me what they were going to do. And then they broke it down into the steps that they would take. I was happy from there…I don't have to worry about this or that.” The investment of time in explaining the process of care for on-reserve horses resulted in greater trust. One person maintained, “Knowing that they're doing everything step by step, because they're in the learning stage is very…reassuring. Normally you'll get the vet that just kind of rushes through and doesn't really explain much.”

The community members’ appreciation for the UCVM program holds strong potential for strengthening relations between the university and Indigenous communities. One horse person was adamant, “keep that partnership going with the university [because] it is really a good partnership. I'm really happy for it.” Another person shared that trust had been earned by program leader JT, “if I do have that full time choice, two or three times a year, guess where I would go…University of Calgary to see this bunch.”

Appreciation for the program extended to pandemic-specific care and the ways in which the program leader (JT) took time to build trust with the horse owners through consistency of care. This included her ongoing visits during COVID when she came without students to decrease the likelihood of spreading COVID to community members. Two horse people described her as “a blessing” while others commented on her work ethic, “just the way she operates. It's a blessing to have her looking after our horses. Everybody's comfortable with her, her relational interaction.” The demonstration of care and compassion built trust, “She does some extra stuff too, like if we needed medicine or anything she'd deliver it to [name of community member]. You know, something that she really cares about. I think she really cares about what's happening here.” Through consistency of care and commitment to the horses and their people, JT has been able to establish good relationships and build trust.

### Reciprocal learning

While the original goal of the community-based rotation was to provide students with the opportunity to learn about equine care through firsthand experiences, the rotation offered the additional benefit of students learning about horses from Indigenous people and perspectives. Indigenous community members noted their role in teaching the students about their culture, including their spiritual connection with the horses. They also shared how longstanding cultural traditions in riding horses are now showcased in contemporary settings, such as rodeos. These reciprocal lessons are an important aspect of the program.

Several horse owners noted appreciation for how the program provided students with an orientation to the community prior to their first visit. One said, “when they come in educated, it's so much more better [for] relationship and understanding. And [it] makes them feel more comfortable [in our community].” The importance of informal conversations in establishing relationships was also mentioned, “Relax, you know, come talk. ‘Cause we love to talk.”

Indigenous community members also viewed the program as an opportunity to further mentor their local youth and to have them consider veterinary medicine as a possible career choice. One said, “hopefully more of our youth and our nation members take that path to veterinary equine services, small animals, just [to be] a veterinarian in general.” Another horse person shared hopes for their own child, “she's going to grade 12 and I was hoping that maybe [I could encourage her to] pursue that direction. To bring that [training] back here to the nation, put a vet on the nation.”

### On-site service provision addressing access issues

A very real, and significant, barrier to accessing veterinary services is the lack of horse trailers for several of the horse people to take their animals to veterinary care—“some people don’t have a horse trailer…but they do have horses.” The veterinary students traveled to each Indigenous community to provide services, this meant that Indigenous horse people without trailers could access services by riding their horses to the designated service area.

### Future connections and recommendations

When prompted, several community members indicated they would like to see veterinary services expanded to include cattle and small animal care, such as dogs and cats, in the community. One horse person explained, “I'd like to see a little more work towards the cattle end of it. You know, we've got cattle here too, and…I think we just doctored them the one time.” Any future service provision of these species would benefit from the lessons learned within the equine-based service-learning project.

## Discussion

**Fig. 2:**
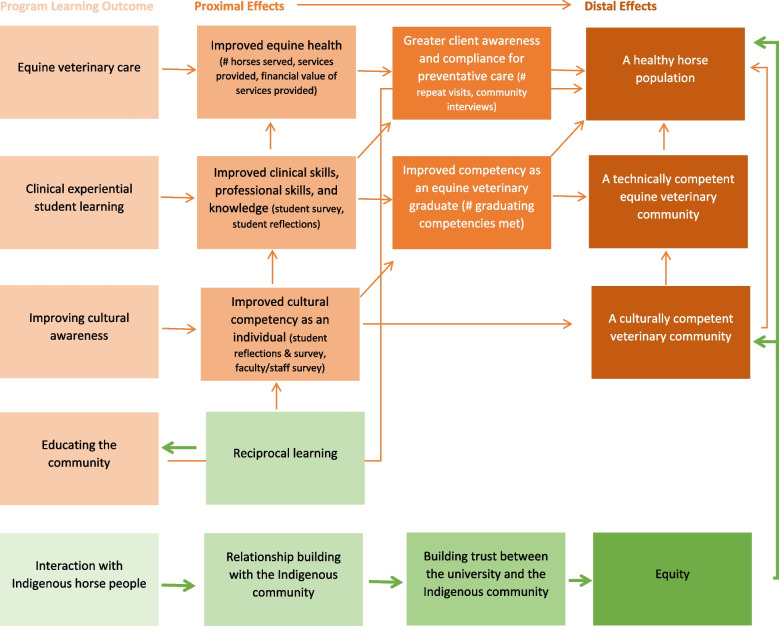
Program impact theory showing actual program effects on proximal and distal outcomes

The findings from this study revealed that the equine service-learning program yielded multiple benefits to a variety of groups. Using program impact theory, it was originally hypothesized that when ethically developed using the “first, do no harm principle,” the existing equine veterinary service-learning initiative produces four main outcomes of the program (Fig. 1): Equine veterinary care, clinical experiential student learning, improving cultural awareness, and educating the community. Convergent, parallel, mixed-methods evaluation using numerical data, student/faculty/staff pre-rotation and post-rotation questionnaires, student reflections, and interviews with community members showed that the program not only yielded the four main outcomes of the program, but led to reciprocal learning and relationship building, which resulted in a trusting partnership and the distal effects of fostering a healthy horse population and competent veterinary community. The additional outcomes discovered during the program evaluation are depicted in Fig. 2.

On an institutional level, the program represents an ethical response to wider calls from the ii' taa'poh'to'p (Indigenous) Strategy implemented by University of Calgary in 2017, the Truth and Reconciliation Commission of Canada’s [2] Calls to Action, and the international United Nations Declaration on the Rights of Indigenous Peoples [3] implemented as law in Canada in June 2021. The creation of this service-learning program was thoughtfully and carefully considered from a “First do no harm” social justice perspective, including lessons delivered by Indigenous community members and experts on the impacts of a colonial past on Indigenous peoples, and in starting the program design from an ethical orientation, the program represented a balanced and equity-driven learning initiative. Community members expressed the importance of this approach through themes found in qualitative analysis, including appreciation for the services and the program leader’s relational ways.

In terms of the impact on equine veterinary care, the program produced quantifiable results, with $136,000 [CAD] veterinary services provided to 50 horse people and 212 horses, including 1100 vaccines, 250 doses of dewormer, 100 dental exams and flotations, and 40 full lameness diagnostic exams and treatment plans. Despite two pandemic years of limited essential services due to cancellation of the student rotation, the value of the services represented significant cost savings to communities that are traditionally limited by financial and geographical restrictions. Pandemic-related shortages in veterinary providers [44], disproportionate impact of the pandemic on Indigenous peoples [42], and the discrimination of Siksika Nation members by local businesses [9, 43] made the complimentary equine veterinary services even more significant.

This service-learning initiative also addressed a significant gap in veterinary student training by way of providing equine-based practica and competence-based skills in the two Indigenous communities involved in the partnership. Participating students were able to exceed the competency requirements expected from a graduating veterinarian by achieving 65 competencies well above the required 51 set out by licensing bodies [11]. Due to the small number of participants (*n* = 4), there were no significant differences in pre-rotation and post-rotation student self-assessments of clinical and professional skills. The sample size required to achieve 0.80 power is 34 participants. Given that an average of four students enroll in the rotation per year, it would take over eight years to amass this data; therefore, this was a formative program evaluation designed to immediately inform the refinement of the program [34]. Despite the shortcomings of quantitative analysis during the study timeframe, qualitative analysis of student reflections showed increased confidence in working on a patient from beginning to end, and practical lessons in case approaches.

Service-learning is reported to result in significant gains in five student outcomes: attitudes towards self, attitudes toward learning, civic engagement, social skills, and academic performance [23]. Convergent, parallel, mixed-methods evaluation of student reflections and student/faculty/staff surveys supported gains in learning and civic engagement. Quantitative data gathered through surveys filled out by students before and after the rotation revealed a tendency towards a change in perception towards social responsibility and engagement (*p* = 0.09), as student data reflected the belief that people from any financial background should own animals (*p* = 0.09). In their reflections, students showed great appreciation for the initiative, each student indicating that the initiative was a worthy use of time and an important part of the curriculum, and indicating they would participate in this type of programming again (strong agreement with statements, *n* = 4).

Civic responsibility in students can be nurtured through classroom instruction, specific interactions with the community, and integration of reflection [45]. Intentional reflection is a core element in effective service-learning that promotes personal, moral, and intellectual development of students [46]. Critical thinking and structured reflection can reinforce community connections and interactions, and this was evident in the qualitative data gathered by way of student reflections on their experiences, which also revealed alignment with the professional commitments set out by the University’s Indigenous Strategy, the TRC Calls to Action [2], and UNDRIP [3]. Those who took part in this service-learning initiative reported a greater valuing of Indigenous worldviews, how they now better understood the impacts of colonialism and systemic racism on Indigenous peoples, and the relationship between systemic racism and veterinary medicine. Importantly, they saw their own role in building authentic relationships with Indigenous communities and community members as a vital part of their professional roles. Because this critical service-learning project intentionally included cultural awareness and social justice training workshops as core components within the curriculum, students were able to attend to and navigate what they were encountering in communities through a new lens of understanding. The training, combined with conversations and experiences with Indigenous community members on reserve, prompted numerous thought-provoking informal “truck” conversations and shared learning during the hours spent traveling to and from the reserve. A clear indicator of this attitudinal shift was evidenced in the student responses to the question, should people who cannot afford animals own them? Their post-program responses reflected a belief in factors beyond solely economic factors in this new understanding. They were also able to see their own role and responsibility in addressing a general lack of knowing and awareness around Indigenous perspectives by others.

From a faculty perspective, the leadership role of an instructor with lived experience and training in social justice and equity-driven responses proved vital to the success of this community-based programming. Community members stressed the importance of the program leader’s relational ways, since she not only considered cultural awareness training as vital to the program design but also understood the need for continuity of care in maintaining “good relations” with the two Indigenous communities she was partnering with in terms of gaining, and earning, trust. The feedback gathered from fellow faculty instructors and staff was less clear in their positioning and reflected some ambiguity in responses. Although there were no statistically significant differences in pre- and post-rotation responses to survey questions, there was a tendency for faculty and staff members to take a firmer stance over the course of their rotation that their beliefs and attitudes are not influenced by their own culture (*p* = 0.10; Table 4). Further studies and commentary would be needed to further investigate this cultural perspective. On the other hand, faculty and staff members seemed to gain appreciation for the need to tailor veterinary care to the unique needs of Indigenous horse people (*p* = 0.10; Table 4), indicating greater alignment to institutional strategic priorities as well as national and international calls to honour Indigenous perspectives [2, 3]. The lack of attitudinal change as compared to students may reflect less interaction with community members, as two of the five faculty and staff members surveyed participated in only one to two of the eight days spent on-site. Further, there was less opportunity for those faculty and staff members who spent less time on-site to process their thoughts through shared learning during conversations with the veterinary team during the commute. Research also shows a decline in prejudice with cohorts who are exposed to racial minorities during formative years when values are formed, meaning that attitude change via generational replacement is larger than attitudinal shifts within a generation [46]. Further research is required in terms of how factors such as cross-generational understandings and early interactions with racial minorities factor into the outcomes of this study.

The hypothesized learning outcomes included equine veterinary care, clinical experiential student learning, improved cultural awareness, and educating the community. One important finding from the student reflections and community interviews was that the education was not one-sided; rather, reciprocal learning occurred. The students reported that what they learned from Indigenous community members around the care and training of horses were invaluable lessons as were the lessons learned relative to the negative colonial impacts on Indigenous peoples.

One additional program learning outcome to those hypothesized was that the interaction with Indigenous horse people led to relationship building, the development of trust, and contributed to equity-building in horse health and a culturally competent community (Fig. 2). Feedback from the Indigenous community members involved in this service-learning program was overwhelmingly positive and in favour of continuation, and even expansion, of this program. Those who received equine veterinary service remarked on how procedures were carefully explained to them by UCVM members and how they felt the program ultimately extended the lives of horses. Members noted how JT was very respectful, solutions-oriented, and committed to doing the best work possible under demanding circumstances and conditions including the pandemic. These comments point to the importance of civic responsibility-based leadership and the importance of mutual understanding and respect among diverse communities [47]. Studies have shown that the most valued leadership characteristics in service-learning are knowledgeable, honest and responsible (personal domain), trusting, dedicated, empowering (interpersonal domain), where the leader demonstrates respectful listening and resourceful attitudes (behavioural domain) [48]. Several Indigenous community members viewed this service-learning program as holding the potential to repair relations with the university; some even noting that they could see their own children being inspired to enter veterinary medicine because of this community-based program. One of the horse people who consistently used these veterinary services worked towards prerequisites for entrance into veterinary school. Notably, the community is beginning to see the value of the program through high school youth and alternative education program involvement. In terms of overall impact on relationship and trust-building, this unique equine service-learning program has brought two worlds closer together than ever before as invitations to meet with local Elders and Chiefs, in formal and community events, continue to be sent out to JT along with plans for the building of a new community building dedicated to this important venture.

The original intention of this service-learning program was to meet gaps in the veterinary curriculum relative to equine skills development in graduating students. The critical social justice orientation of the service-learning program first developed by JT, and later supported by YPP and PD, yielded positive benefits beyond these initial expectations. From positive and high-quality student learning outcomes to that of recognition of positive impact by Indigenous community members, this initiative demonstrated the results of careful and ethically considered pre-planning by a faculty leader who recognized the importance of humility, respect, and relationship-building in the overall planning and delivery of this program. The principle of reciprocity whereby students were gifted with as much as they gave was one of the major highlights, and unexpected outcomes, of this service-learning initiative.

Findings from interviews with community members include possible future directions for the program, including small animal services, production animal services, and further considerations for limited horse transport. Some community ideas have already been implemented, such as mentorship opportunities for high school students to shadow veterinary students during the program.

Limitations of the study include the small sample size (student participants *n* = 4; faculty/staff participants *n* = 5), which did not achieve statistical power for quantitative analysis and limited our ability to detect true effects. This was mitigated using student reflections for qualitative thematic analysis and the use of the program evaluation is formative. There was also potential for bias in community interviews due to the desire to not lose veterinary services that have substantial financial value. This was mitigated by using a third party (research assistant) who independently approached community participants, offered anonymity, and offered financial compensation in exchange for the interview.

This is the first-documented equine veterinary service-learning initiative in Indigenous communities embedded in a veterinary curriculum. The success of this program and achievement of five program learning outcomes with many positive proximal and distal effects, represents an opportunity for emulation at many veterinary institutions. It is essential to note that the “first, do no harm principle” was carefully considered at each step of the program, with considerations for positionality and anti-racism. Importantly, this program evaluation specifically examined impact rather than intent, since well-intentioned social programs do not necessarily lead to better outcomes and can even result in negative repercussions [31]. As such, the program was approached with emphasis on partnership and reciprocity, overt racial justice and diversity training, sustainability and longevity, and acknowledgement that the program operates on an invitation-only basis, renewed annually on mutual consent by each party. Finally, program evaluation is a critical step in evaluating impact and informing program improvements. 

## Conclusion

The partnership between Tsuut’ina and Siksika Nations and University of Calgary signifies the first-documented equine veterinary service-learning initiative in Indigenous communities embedded in a veterinary curriculum. The outcomes of the program were systematically evaluated using a convergent, parallel, mixed-methods evaluation that led to the discovery of benefits in reciprocal learning and relationship-building with the Indigenous community in addition to hypothesized benefits from provision of equine veterinary care, clinical experiential student learning, improving cultural awareness, and educating the community. As a unique learning initiative within the UCVM community and well beyond, this critical service-learning program holds several wise practices that carry relevance for anyone wishing to replicate its design. First and foremost, veterinary faculty members must acknowledge the “first, do no harm” principle, embodied by the need to balance and to “right relations” with Indigenous communities, community members, and those who hold a special relationship as caretakers of their four-legged relatives. Understanding the ways in which colonial injustices impact the contemporary lived experiences of the First Peoples in Canada is essential but also vital is the recognition by faculty members of how they have personally been advantaged by the same systems. This is not easy learning and is often viewed by non-Indigenous instructors, and students, as outside the purview of formal educational structures that have either ignored or dismissed Indigenous worldviews. The need to work with, and not on or for, Indigenous communities is another essential learning gained from this program as institutions and instructors learn to value Indigenous knowledge systems on the same level as Eurocentric curricular outcomes. The principle of reciprocity seeks equitable relations and requires the sharing of power and control through a stance of mutual respect and recognition.

## Data Availability

The datasets generated during and/or analysed during the current study are available from the corresponding author on reasonable request.

## Footnotes

1 The term “horse person” is used throughout this article to honour Indigenous ways of knowing that position animals as non-human relatives, as compared to colonial modes of relation that position animals as possessions and thus deems the relationship as “horse owner”[49].
